# Ethanol Alters the Neurexin Landscape in Human Neuroblastoma Cells

**DOI:** 10.1111/acer.70289

**Published:** 2026-04-12

**Authors:** Clara C. Lowe, Kip D. Zimmerman, Rita Cervera‐Juanes

**Affiliations:** ^1^ Department of Translational Neuroscience, School of Medicine Wake Forest University Winston‐Salem North Carolina USA; ^2^ Center for Precision Medicine, School of Medicine Wake Forest University Winston‐Salem North Carolina USA; ^3^ Department of Internal Medicine, Section of Molecular Medicine, School of Medicine Wake Forest University Winston‐Salem North Carolina USA; ^4^ Department of Biostatistics and Data Science, Section of Molecular Medicine, School of Medicine Wake Forest University Winston‐Salem North Carolina USA

**Keywords:** alcohol, alternative splicing, neurexin, parvalbumin, synaptic plasticity

## Abstract

**Background:**

Neurexins (NRXNs) are presynaptic adhesion molecules essential for synaptic organization and the regulation of excitatory–inhibitory balance. The molecular diversity of NRXNs arises from alternative promoters and splicing, particularly at splice site 4 (SS4), which dictates ligand binding. Dysregulation of NRXNs has been linked to substance use disorders, but it remains unclear how the expression of NRXN isoforms responds to physiologically relevant amounts of ethanol.

**Methods:**

Human IMR‐32 neuroblastoma cells were maintained in an undifferentiated (UnDiff) state or differentiated (Diff) with trans‐retinoic acid (tRA) to promote an enrichment in parvalbumin (PV) expression. Cells were exposed to physiologically relevant ethanol concentrations (0, 7, or 35 mM) in vapor chambers. Quantitative polymerase chain reaction (qPCR) quantified mRNA levels of major NRXN transcripts (*NRXN1*, *NRXN2*, and *NRXN3*) and SS4 variants (+SS4, −SS4). Immunocytochemistry (ICC) was used to measure protein expression and overlap with neuroligin2 (NLGN2) and PV.

**Results:**

Differentiation increased basal expression of several NRXN transcripts, including *NRXN2α*, *NRXN2 +SS4*, *NRXN3α*, *NRXN3β*, and *NRXN3* −*SS4*. In Diff cells, ethanol‐induced dose‐dependent downregulation of *NRXN2α*, *NRXN3α*, *NRXN3β*, and *NRXN3* −*SS4* transcripts, while *NRXN1* remained stable. In Diff cells, ICC confirmed isoform‐specific protein reductions without changes in other markers (Tuj1 and PV). NRXN3β decreased at 7 and 35 mM; and NRXN1 and NRXN2 at 35 mM. Ethanol significantly reduced overall expression of NRXN3β at 7 and 35 mM; and NRXN1 and NRXN2 at 35 mM, along with NRXN3β‐NLGN2 spatial overlap and NRXN1, 2, and 3β signal within PV‐positive cells, indicating targeted disruption of inhibitory synaptic organization.

**Conclusions:**

Physiologically relevant ethanol exposure alters NRXN expression in an isoform‐, splice site‐, and differentiation‐dependent manner, prominently affecting NRXN3 and the SS4 site. These coordinated transcriptional and proteomic changes suggest that ethanol perturbs NRXN3β‐NLGN2 interactions and inhibitory synapse stability, revealing a molecular pathway where alcohol may compromise cortical network excitatory–inhibitory balance.

## Graphical abstract

It is currently unknown how ethanol exposure alters the landscape of the synaptic hubs, neurexins. Using IMR32 cells differentiated toward parvalbumin‐like neurons, we demonstrated an ethanol dose‐dependent, transcript and isoform‐specific loss of neurexins. Specifically, neurexin3β and its binding partner neuroligin2 are particularly sensitive to ethanol's effects.

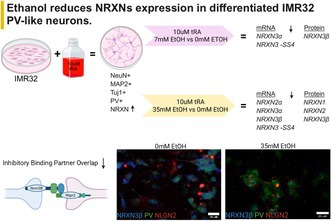

## 1 Introduction

Neurexins are a family of presynaptic cell adhesion molecules critical in neuronal communication and plasticity throughout the central nervous system (CNS) (Reissner et al. 2013). Encoded by three genes—*NRXN1*, *NRXN2*, and *NRXN3*—neurexins exhibit a remarkable structural diversity, giving rise to multiple isoforms through alternative splicing and the use of alternative promoters. These isoforms include three main classes: alpha (α), beta (β), and the less characterized gamma (γ), which differentially interact with a variety of postsynaptic proteins to mediate synaptic organization, specification, and signaling (Sudhof 2017; Gomez et al. 2021; Bang and Owczarek 2013; Boxer and Aoto 2022).

Among the six different alternative splicing sites, splice site 4 (SS4) acts as a molecular switch driving synaptic identity (Boucard et al. 2005; Aoto et al. 2013; Roppongi et al. 2020) and conferring synaptic specificity (Treutlein et al. 2014; Liakath‐Ali and Sudhof 2021). Inclusion (+SS4) or exclusion (−SS4) of SS4 modulates binding affinity to different postsynaptic proteins, such as neuroligins (NLGNs), leucine‐rich repeat transmembrane proteins (LRRTMs), cerebellins (CBLNs), and dystroglycan (Reissner et al. 2013; Aoto et al. 2013; Treutlein et al. 2014; Trotter et al. 2023; Boxer and Aoto 2022; Boucard et al. 2005; Gomez et al. 2021). More specifically, certain combinations of NRXN isoforms with particular postsynaptic binding partners are selectively enriched at excitatory or inhibitory synapses. For instance, in parvalbumin (PV) inhibitory synapses, −SS4 isoforms preferentially bind to NLGN2 (Liakath‐Ali and Sudhof 2021), which is enriched at inhibitory synapses, promoting the formation of stable trans‐synaptic complexes that regulate GABAergic transmission (Graf et al. 2004). In contrast, +SS4 isoforms reduce affinity for NLGN2 but enhance interaction with CBLNs, particularly CBLN4, which modulates short‐term plasticity, neurotransmitter release probability (Luo et al. 2021), and the fast‐spiking precision characteristic of PV interneurons (Roppongi et al. 2020). In the cortex, this splicing‐dependent ligand switch allows PV interneurons to diversify their trans‐synaptic signaling mechanisms, thereby fine‐tuning inhibitory output and cortical circuit integration (Trotter et al. 2023). Given their role as dynamic organizers of synapses, the disruption of NRXN isoform expression or alterations in SS4 splicing have been associated with neurodevelopmental disorders (Cao and Tabuchi 2017). In addition, dysregulation of NRXNs may also contribute to the alterations in synaptic function widely reported in substance use disorder (Hishimoto et al. 2007; Sasabe and Ishiura 2010; Stoltenberg et al. 2011; Ferranti et al. 2022), including alcohol use disorder (AUD). Chronic ethanol is known to disrupt the excitatory/inhibitory (E/I) balance in AUD, through favoring an excess in excitatory signaling along with a decrease in inhibitory tone. In the medial frontal cortex of humans and across various brain regions throughout the mesolimbic system in rats, chronic ethanol consumption increased basal extracellular glutamate concentrations and reduced glutamate clearance (Ding et al. 2012, 2013; Melendez et al. 2005). In addition, reduced GABA‐A receptor (a direct binding partner of NRXN3β [Zhang et al. 2010]) abundance in the frontal cortex of ethanol self‐administering nonhuman primates (Hemby et al. 2006) and in the cerebral cortex of ethanol‐consuming rats (Mhatre et al. 1993) has been reported. As such, investigating NRXN transcript/isoform and splicing adaptations—particularly at SS4—in response to ethanol exposure provides a valuable framework for uncovering the molecular mechanisms that may underlie addiction‐related neuroplasticity (Zhang et al. 2023; Smith et al. 2025). To begin addressing this gap, we used human IMR32 neuroblastoma cells untreated or treated with trans‐retinoic acid (tRA) to promote differentiation toward cortical PV‐like interneurons. These cells were then exposed to physiologically relevant doses of ethanol (7 and 35 mM) (Thierauf‐Emberger et al. 2020) prior to molecular profiling. With this highly controlled in vitro model, we assessed the expression of all major NRXNs (NRXN1, NRXN2, and NRXN3) at both the mRNA and protein levels following ethanol exposure in undifferentiated (UnDiff) and tRA‐differentiated (Diff) conditions. This approach enables a mechanistic dissection of how ethanol modulates all major NRXN isoforms within PV‐like inhibitory cells (Ferguson and Gao 2018; Hu et al. 2014).

## 2 Methods

### 2.1 Cell Culture

Human neuroblastoma cells (IMR‐32, ATCC) were cultured in Eagle's Minimum Essential Medium supplemented with 10% fetal bovine serum (FBS), 100 U/mL penicillin, 100 μg/mL streptomycin, and 0.004% gentamycin at 37°C under 5% CO₂/95% air, with medium replaced twice weekly. Upon reaching confluence, cells were rinsed with Dulbecco's phosphate‐buffered saline (DPBS; Ca^2+^‐ and Mg^2+^‐free) and detached using trypsin–EDTA (0.25% trypsin and 0.02% EDTA). Trypsin was blocked with complete growth medium. For ICC, ~2,500–5,000 cells/well were seeded into 8‐well Culture Slides (catalog no.: 354118). For all other analyses, ~25,000 cells/well were seeded into 6‐well plates to conduct the experiments described below. To avoid variation across replicates, similar passage numbers (P16‐P22) were used across all replicates.

### 2.2 IMR32 Differentiation Using tRA

Based on prior literature (Harasym et al. 2017; Haussler et al. 1983; Sidell et al. 1983), we tested a range of tRA (Sigma Chemical Co., St. Louis, MO) concentrations: 10.00, 12.50, and 15.00 μM (tRA dissolved in DMSO), on its ability to induce differentiation of IMR32 cells. Cells were assessed at 48, 96, 120, and 144 h post‐treatment for neurite outgrowth and cell viability. Medium containing fresh tRA was replaced twice weekly.

### 2.3 Exposure of IMR32 Cells to Physiologically Relevant Concentrations of Ethanol

Ethanol concentrations for in vitro exposure were selected based on physiologically relevant values reported in a human study using magnetic resonance spectroscopy (MRS) to quantify ethanol distribution in the brain and serum following controlled alcohol intake (Thierauf‐Emberger et al. 2020). A peak ethanol concentration of 0.68 g/L in the frontal cortex (equivalent to ~14.80 mM when normalized to brain water content) was observed when participants reached a serum ethanol concentration of ~0.99 g/L, corresponding to an administered dose of ~0.80 g/kg body weight. We based our in vitro concentrations on the average serum‐to‐brain ratio (~1.67) derived from values reported across the frontal cortex. We then applied the reported serum‐to‐blood conversion factor of 1.24 (to get serum g/L in g/kg) and estimated that a brain concentration of 35 mM, corresponding to ~58.50 mM in serum (~269 mg/dL), equates to ~0.22% BAC. A lower concentration of 7 mM in brain corresponds to ~11.70 mM in serum (~53.90 mg/dL), and ~0.04% BAC. Based on these physiologically grounded values, IMR32 cells were treated with 0 mM (control), 7 mM (low), and 35 mM (high) ethanol to model sub‐intoxicating and intoxicating exposures consistent with behavioral and cognitive changes observed in human populations.

**FIGURE 1:**
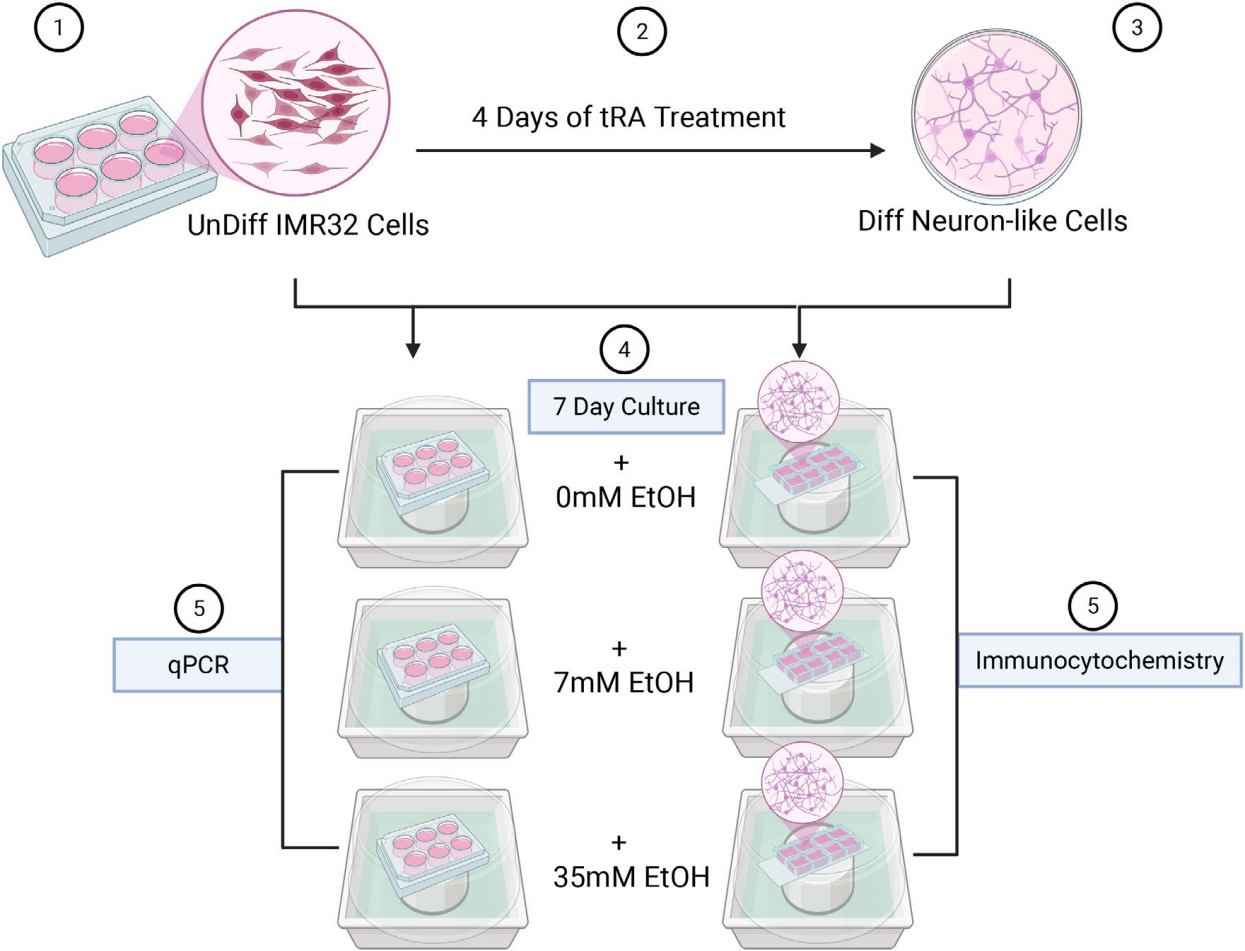
Experimental design for assessing ethanol‐associated molecular changes in differentiated human neuroblastoma cells. (1–3) Human IMR32 neuroblastoma cells were cultured and differentiated using all‐trans retinoic acid (tRA; 10 μM), inducing a differentiated neuron‐like phenotype. (4) Following differentiation, cells were exposed to 0 mM, 7 mM, or 35 mM ethanol for 7 days in chambers to model physiologically relevant alcohol exposure. (5) Downstream molecular analyses included qPCR for transcript‐level changes (including splice transcript resolution), and immunocytochemistry to assess protein expression patterns.

For the mRNA analysis, IMR32 cells were seeded in triplicates (biological replicates) and divided into two experimental groups: one group underwent an initial 4‐day differentiation phase with 10 μM tRA to promote a cortical neuron‐like phenotype, whereas the other group was treated with vehicle and remained as UnDiff cells. After the initial tRA/vehicle treatment, both groups were cultured under varying ethanol concentrations (0, 7, and 35 mM) in ethanol vapor chambers while supplementing the culture medium with tRA/vehicle (Figure 1). Briefly, the 6‐well plate containing the cells was positioned on a solid platform in the middle of a Tupperware chamber containing the desired ethanol concentration (diluted in water). The chamber was sealed with cling wrap, and perforated to allow gas exchange while minimizing ethanol vapor loss. Half of the culture media was replaced every third day, with the Diff group receiving fresh tRA and ethanol, whereas the UnDiff group received fresh ethanol and vehicle. Media from both the Diff and UnDiff cultures were sampled over the course of the ethanol exposure treatment to confirm stable ethanol content, ~9.23–9.31 mM for the 7 mM samples, and ~29.3–33.3 mM EtOH for the target 35 mM concentration (Carolina Liquid Chemistries Corp. Etha Rgt. Kit BL‐421). For immunocytochemistry (ICC) Following, cells were treated the same.

### 2.4 Live/Dead Viability Assay

Cell viability of Diff IMR32 cells following ethanol treatment was assessed using the LIVE/DEAD Viability/Cytotoxicity Kit (Thermo Fisher Scientific). Following ethanol treatment, medium was replaced with a staining solution (5 μL Calcein AM (Component A) and 20 μL Ethidium Homodimer‐1 (Component B) in 10 mL DPBS) and incubated at room temperature (20°C–25°C) for 30 min in the dark. Live cells exhibited green fluorescence from Calcein AM, whereas dead cells exhibited red fluorescence due to Ethidium Homodimer‐1 labeling. Quantitative analysis was performed using ImageJ by thresholding the green and red channels to calculate the ratio of live to dead cells, with three biological replicates per condition.

### 2.5 RNA Extraction and cDNA Synthesis

At the end of the tRA/vehicle and ethanol treatment (11 days total, 4 days of differentiation followed by 7 days of ethanol treatment under differentiating conditions), total RNA was isolated using the RNeasy Mini Kit (Qiagen) according to the manufacturer's protocol. RNA concentration and purity were determined using a Nanodrop spectrophotometer, and RNA was stored at −80°C until use.

Synthesis of complementary DNA (cDNA) was performed with 100 ng of total RNA using the SuperScript IV Reverse Transcriptase system (Invitrogen, Vilnius, Lithuania) per manufacturer's instructions. The resulting cDNA was either used stored at −20°C until use.

### 2.6 Quantitative PCR (qPCR)

qPCR was performed using GoTaq qPCR Master Mix (1×, Promega, Madison, WI, USA) with forward and reverse primers at 400 nM each (primer sequences listed in Table 1) and template cDNA (10 ng). Thermal cycling conditions included an initial denaturation at 95°C for 2 min followed by 40 cycles at 95°C for 15 s and 59°C for 30 s. Optimization of primer concentrations and annealing conditions was performed to ensure unique peaks in the melting curve. Expression data were normalized to the housekeeping gene *B‐ACTIN*, based on NormFinder (Andersen et al. 2004) analysis using *B‐ACTIN*, *PGK1*, and *GAPDH* as potential housekeeping genes. Relative quantification was calculated by the ΔΔCt method. To determine differences between UnDiff cells and Diff, in an alcohol‐naïve state, fold change was computed using the mean of the 0 mM UnDiff group as the calibrator for each transcript (i.e., ΔΔ*C*_t_ = Δ*C*_t(7mM or 35mM)_ – mean Δ*C*_t(UD, 0mM)_). Therefore, in these comparisons, the fold change of 0 mM UnDiff would be 1; anything above 1 would indicate a relative upregulation compared with 0 mM, whereas anything below 1 would indicate a relative downregulation. Within each differentiation group (UnDiff or Diff), fold change was computed using the mean of the 0 mM group as the calibrator for each transcript (i.e., ΔΔ*C*_t_ = Δ*C*_t(7mM or 35mM)_ – mean Δ*C*_t(0mM)_).

**TABLE 1:** List of primers and their sequences used for real time polymerase chain reaction.

Primer target	Direction	Sequence
Beta Actin	Forward	AATGTGGCCGAGGACTTTGAT
Beta Actin	Reverse	AGTGGGTGGCTTTTAGGAT
NRXN1α	Forward	AACGCAAATCACCGCCGGA
NRXN1α	Reverse	GCCTTCTTTGGCATGTACAAG
NRXN1β	Forward	GCCTATTGCAATCTACAGGTC
NRXN1β	Reverse	AACCTATGGCCAGTCTGTCT
NRXN1γ	Forward	GCCAGACAGACATGGATATGAG
NRXN1γ	Reverse	TGAGGCCACAAGGATGTCAT
NRXN1 +SS4	Forward	AATGGCTACTCGACAAAGGG
NRXN1 +SS4	Reverse	GCGATGTTGGCATCGTTTTC
NRXN1 −SS4	Forward	TACCCTGCAGGGCGTCAGCT
NRXN1 −SS4	Reverse	CGGCTGCCATATTCAGAACT
NRXN2α	Forward	ACTGAAGAGTCCTGTGCCAA
NRXN2α	Reverse	CCTTCCCAAAGATGTATGTGG
NRXN2β	Forward	AGCAACACTTCGCTGCCTC
NRXN2β	Reverse	GAAGGGAAGCAAATCCGGGTG
NRXN2 +SS4	Forward	CTTTGATAACGAGCGCCTGG
NRXN2 +SS4	Reverse	GCCTGGCTGTTGAAGATGGT
NRXN2 −SS4	Forward	TACCCGGCAGGCCGCCA
NRXN2 −SS4	Reverse	CACCTTGAGCCCATTGTAGT
NRXN3α	Forward	ACCCAGTACCACCTGCCAGGAA
NRXN3α	Reverse	TCATTGCACTGGTTTCCAGAA
NRXN3β	Forward	GCATCACTCAGTGCCTATTTC
NRXN3β	Reverse	CAAGATGCCATCCTTCACAG
NRXN3 +SS4	Forward	GCAACACTGATAATGAACGC
NRXN3 +SS4	Reverse	TGCGCCTGAGTGTTGAAGAT
NRXN3 −SS4	Forward	ATTATCCTACAGGCCGGCAG
NRXN3 −SS4	Reverse	TTCTCAGCCGCCATGTTCAG

### 2.7 Immunocytochemistry (ICC)

We validated tRA‐induced neuronal differentiation in our culture system using cell type‐specific markers (NEUN, TUJ1, MAP2, and PV). We also quantified ethanol‐associated changes in axons (TUJ1), PV‐positive cells, NRXN isoforms, and the overlap of NRXNs with their inhibitory binding partner NLGN2 after tRA differentiation and ethanol exposure.

Following ethanol exposure as described above, Undiff and Diff treated cells were fixed in 4% paraformaldehyde (PFA) for 10 min at room temperature, permeabilized with PBS containing 0.1% Tween‐20 for 10 min and blocked with 10% donkey serum in PBST (0.05% Tween‐20) for 1 h. Primary antibodies were applied at the following dilutions: 1:800 PV (catalog no.: PA5‐143579, Invitrogen, Rockford, IL, USA), 1:100 Beta 3 tubulin (TUJ1), 1:800 microtubule‐associated protein 2 (MAP2), 1:50 NLGN2, 1:50 NRXN1, and 1:50 NRXN2, 1:400 NRXN3β (catalog no.: A5‐101708, Invitrogen, Rockford, IL, USA) and 1:30 NRXN3α (catalog no.: AF5269, R&D Systems, Minneapolis, MN, USA) in PBS containing 0.05% Tween‐20 and 5% donkey serum, and incubated overnight at 4°C. After washing, secondary antibodies conjugated to Alexa fluorophores (e.g., catalog nos.: A48257, A48258, A78948, A31573, A10042, and A21099; Invitrogen) were applied, along with nuclear stains (Hoechst, catalog no.: H3569; Invitrogen, Eugene, OR, USA at 1:10,000, or DRAQ5, catalog no.: 62251; Thermo Scientific, Rockford, IL, USA at 1:3000). Fluorescent images were captured using a Leica DMi8 microscope, and quantification was performed using ImageJ and our customized Python script executed in Google Colab using OpenCV, NumPy, and Matplotlib, as follows. Single‐channel PNG images were generated for each channel, with an optional pre‐merged composite image. Intensities were normalized and perceptual contrast enhanced via gamma correction with *γ* = 0.8, followed by clipping to 8‐bit range. Binary segmentation masks were then derived for each channel by fixed global thresholds. For each mask, total positive area was computed as the fraction of suprathreshold pixels within the field (reported as % area positive for each signal). To visualize segmentation fidelity, panels showing the original grayscale image, its binary mask, and the pairwise overlap (maskwise AND) were rendered. A composite heatmap was generated by summing per‐pixel mask memberships and color‐coding voxels belonging to exactly two, three, or four channels. For quantitative comparisons across channels, a denominator “merged” area was defined either from the provided merged mask or, when absent, as the pixelwise union (bitwise OR) of all individual channel masks. For each channel, total % area was expressed relative to the merged area (merged = 100%). Pairwise overlap with a target channel (first pass: NRXN; second pass: other marker) was computed as the area of the bitwise AND between the target and each comparison mask, reported both as (1) overlap % relative to the merged area (spatial prevalence within the multi‐label scene) and (2) overlap % relative to the target's own area (fraction of target signal coincident with the comparison marker). For each marker, we report the total area (% over merged), overlap with the target (% over merged), and target‐referenced overlap (% over target). A diagnostic “total overlap” image (hot colormap) was also displayed to highlight regions where multiple channels coincided. Parameters (resize, γ, and thresholds) were held constant across images within a batch to ensure comparability.

Analyses were limited to isoforms for which validated antibodies were available (NRXN1, NRXN2, NRXN3α, and NRXN3β), and thus are not intended to specifically discriminate across all existing NRXN isoforms.

As we could only investigate four proteins per well (given limitations in the number of filters in our microscope), and to maximize the amount of output data per well, we used different combinations of antibodies per well. As a result, NRXN3α, NRXN3β, and PV were analyzed in more wells than other markers (TUJ1, NRXN1, NRXN2, and NLGN2), and therefore, we have more biological replicates for them. Per each well, we captured and analyzed at least three non‐overlapping fields (40×; that were treated as technical replicates). The technical replicates were averaged, and the mean ± standard deviation values were used for statistical analysis. The specific number of biological replicates is displayed in the corresponding figure legends.

### 2.8 Statistical Analyses

A Levene's test (alpha value of 0.05, F effect type, including outliers) and a Shapiro–Wilk test were used to assess the homogeneity of variance and normality of the data prior to statistical analysis (Levene 1960; Shapiro and Wilk 1965). When assumptions of homogeneity of variance or normality were violated, non‐parametric methods, such as the Kruskal–Wallis (Kruskal and Wallis 1952) test, were employed. If sphericity was not met, as determined by Mauchly's (Mauchly 1940) test, adjustments were made using the Greenhouse–Geisser correction (Greenhouse and Geisser 1959). Gene expression differences in IMR32 UnDiff and Diff cells were analyzed using several statistical analyses depending on the question. The live:dead ratio assays across ethanol concentrations were analyzed using a Brown‐Forsythe and Welches ANOVA test, followed by a post hoc Dunnets T3 analysis. Differences at 0 mM ethanol depending on differentiation status were analyzed using an unpaired Student's *t*‐test. For interrogating differences depending on ethanol concentration and differentiation status, each NRXN transcript was analyzed with two‐way ANOVA (*α* = 0.05) with a full‐effects model, including the interaction term using the two fixed factors of differentiation status (UnDiff vs. Diff) and ethanol concentration (7 mM vs. 35 mM), followed by a Sidak's post hoc analysis. Within UnDiff and Diff groups, to probe ethanol‐induced differences (0 mM vs. 7 mM and 0 mM vs. 35 mM) in NRXNs, one‐way ANOVA followed by Dunnets post hoc (familywise *α* = 0.05) analyses were used. The ICC data were analyzed with a two‐way ANOVA with fixed factors: protein (six levels: NRXN1, NRXN2, NRXN3α, NRXN3β, PV, TUJ1, and NLGN2) and ethanol dose (two levels: 0, 7, and 35 mM), including the interaction (12 degrees of freedom (df)) between ethanol dose and NRXN isoform. To test dose‐specific differences, we used Tukey post hoc comparisons. When computing tests with the two‐sided Student's *t*‐tests, if normality was violated, data were log‐transformed. Means back‐transformed for display. All statistical results were presented as mean ± SEM, and significance was set at a *p* value of less than 0.05 for differences among groups after multiple comparison correction.

## 3 Results

### 3.1 Selection of the tRA Concentration to Induce Differentiation

**FIGURE 2:**
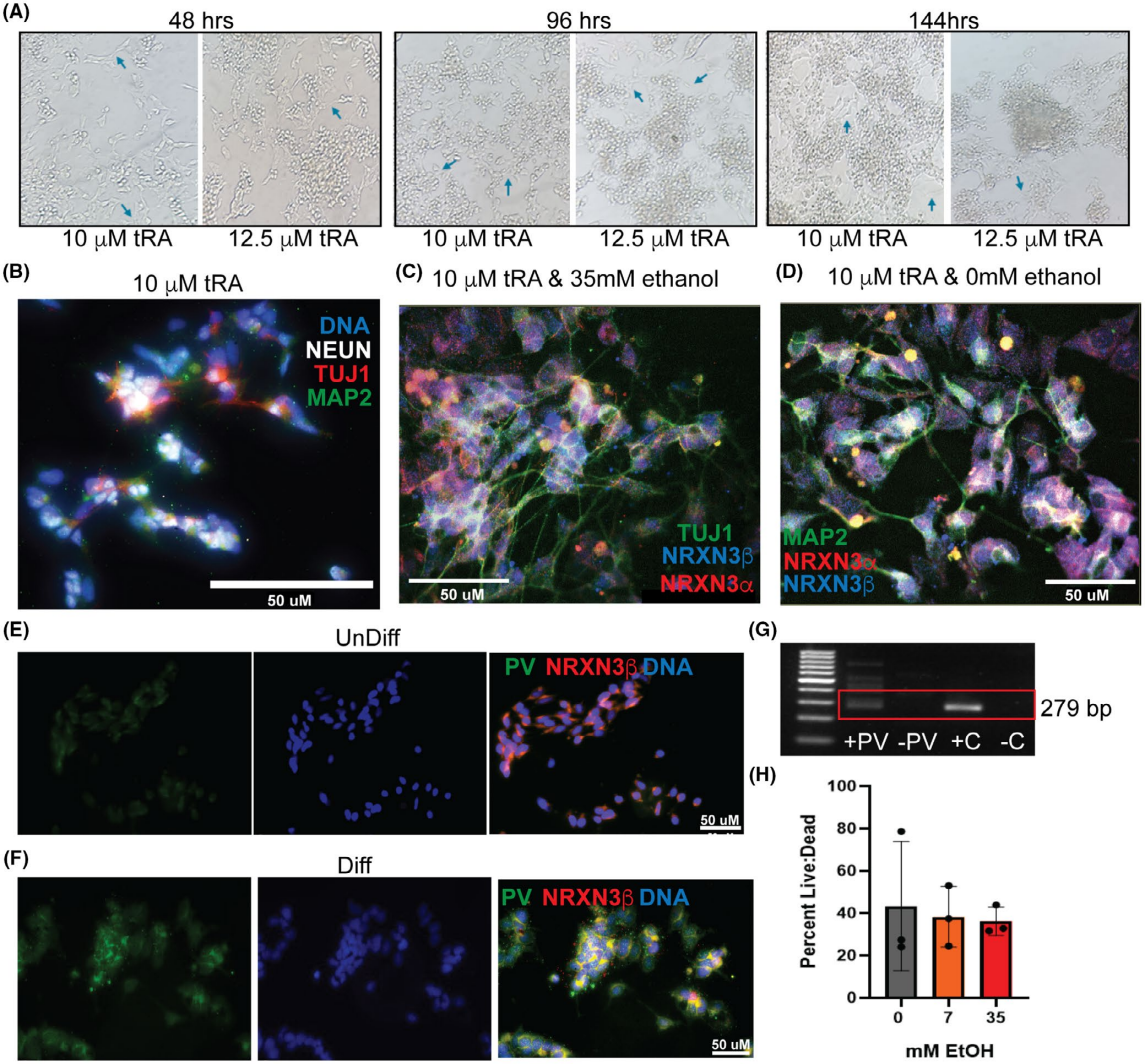
Differentiated IMR32 cells exhibit more mature post mitotic neuronal features following all‐trans retinoic acid treatment without inducing cytotoxicity. (A) Representative bright field images showing IMR‐32 cell morphology over 48, 96, and 144 h of differentiation with either 10 or 12.5 μM all‐trans retinoic acid (tRA), demonstrating progressive neurite outgrowth indicative of neuronal differentiation. Cell death and detachment was evident at 12.5 μM tRA concentration at later time points. (B) 40× Immunofluorescent (IF) labeling of axons using βIII‐TUBULIN (TUJ1, red), dendrites (MAP2, green), Neuronal nuclei (NeuN, white), and DNA (Dapi, blue) after 4 days of differentiation with tRA (10 μM). (C) IF labeling of TUJ1 (green), NRXN3β (blue), and NRXN3α (red) after 4 days of differentiation with tRA (10 μM), followed by 7 days of continued culture under 35 mM ethanol‐exposed conditions. TUJ1 highlights neuronal lineage, whereas NRXN signals localize to both somatic and neuritic compartments. (D) IF co‐labeling of MAP2 (green), NRXN3β (blue), and NRXN3α (red) in differentiated IMR32 cultures exposed to 0 mM EtOH for 7 days. MAP2 labeling delineates neuronal perikaryal and dendritic architecture, revealing the spatial distribution of NRXN isoforms within dendritic processes. (E–F) 40× magnification of undifferentiated IMR32s (E) and differentiated IMR32s (F)—PV (green), NRXN3β (red) and DNA (blue). (G) PCR analysis detects an upregulation in PV in differentiated cells. As a positive control, we used cDNA from human nucleus accumbens (+C). 100‐bp ladder shown on the left, cDNA negative control on the far right. (H) No significant differences were observed in the ratio of live to dead cell percent area across ethanol treatment groups.

Although 12.5 and 15 μM of tRA‐induced neurite extension as observed under light microscopy, they were also associated with increased cell death, particularly beyond 96 h of tRA treatment. In contrast, 10 μM tRA consistently promoted robust neurite outgrowth with minimal cytotoxicity, even at the 96 and 144‐h timepoints (Figure 2A). After ~4 days of 10 μM tRA treatment, cells displayed neurite growth and expressed markers of neuronal differentiation, including NEUN, MAP2, and TUJ1 (see representative images in Figure 2B–D). In agreement with the literature (Donato et al. 2013), following tRA treatment, there was also an enrichment in neuronal (NEUN +) cells expressing PV (Figure 2B,E,G), which were very low to absent in UnDiff (Figure 2F), indicative of an enrichment toward PV specification after tRA treatment. This balance of cell survival and morphological differentiation informed our decision to use 10 μM as the optimal working concentration for downstream assays.

### 3.2 Selection of Ethanol Concentrations

Using 10 μM of tRA, we ran a preliminary analysis and tested the effects of a wide range of ethanol doses: 0, 7, 14, 21, 28, 35, and 70 mM for 7‐, 10‐, and 14‐days on cell viability and *NRXN* expression. Treatment with 70 mM ethanol (1.36 g/L in the brain, ~0.44% BAC) led to global cell death, indicating that the use of supraphysiological ethanol concentrations leads to generalized cell death, at least in this cell line, and would obscure biologically meaningful outcomes of the non‐toxic effects of ethanol. Among all the other doses, the 7 mM (low) and 35 mM (high) doses were chosen for downstream analyses as they represent the extremes of physiological brain levels of ethanol (as described above), capturing features of intoxication and below‐intoxication levels, with no statistically significant differences in live:dead cell ratio (*F*_(df=2)_ = 0.1, p_Brown‐ForsytheANOVA_ = 9.0 × 10^−1^) (Figure 2H).

### 3.3 Low Ethanol Exposure Altered the Expression of Specific *NRXN* Transcripts in UnDiff IMR32 Cells

**FIGURE 3:**
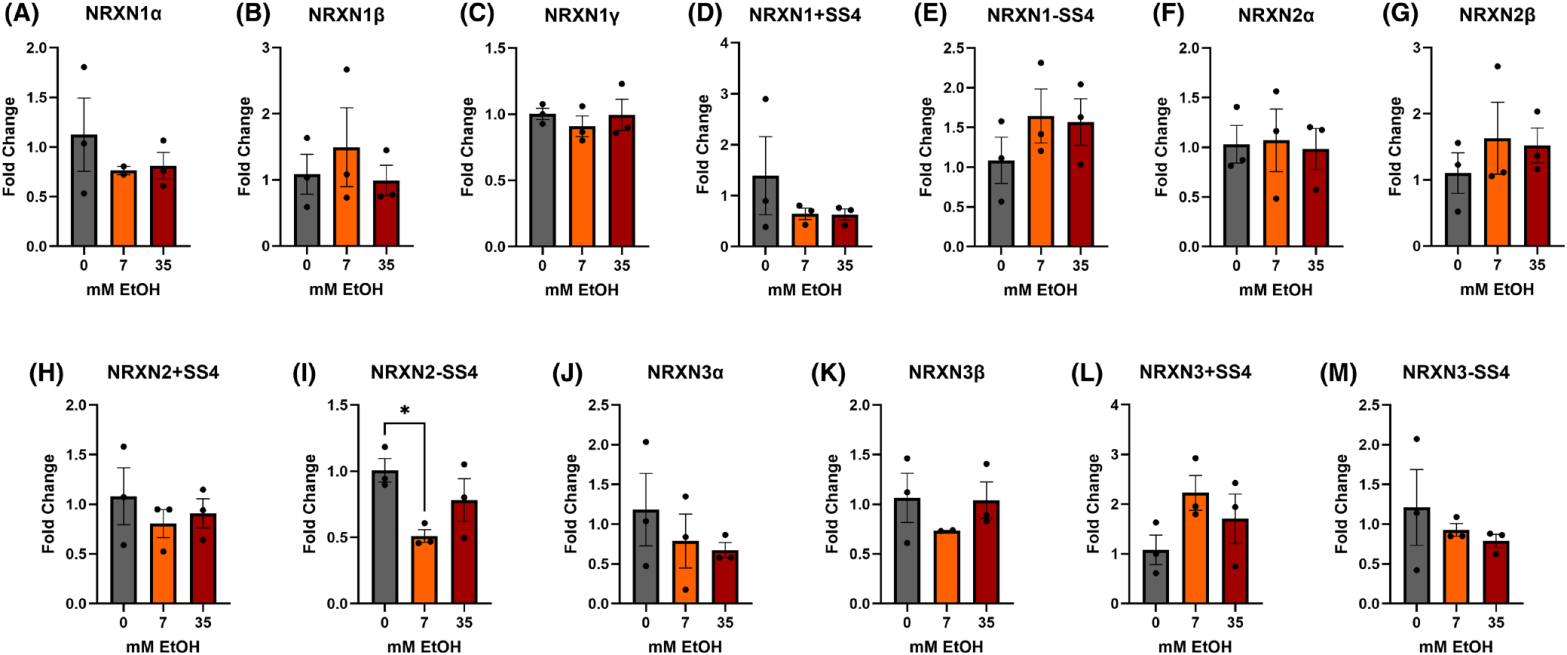
Quantitative PCR analysis of neurexin (NRXN) mRNA expression following 7 days of ethanol exposure in undifferentiated IMR32 cultures. qPCR fold change (*y*‐axis) for neurexin transcripts and SS4 splice variants—(A) NRXN1α, (B) NRXN1β, (C) NRXN1γ, (D) NRXN1 +SS4, (E) NRXN1 −SS4, (F) NRXN2α, (G) NRXN2β, (H) NRXN2 +SS4, (I) NRXN2 −SS4, (J) NRXN3α, (K) NRXN3β, (L) NRXN3 +SS4, (M) NRXN3 −SS4—after exposure to 0, 7, or 35 mM EtOH. Bars show mean ± SEM; points denote biological replicates. *p* values reflect one‐way ANOVA with post hoc Dunnett's correction for multiple comparisons as described in methods.

qPCR analysis revealed that all 13 *NRXN* transcripts were expressed in UnDiff cells, regardless of the ethanol concentration used (0 mM, 7 mM, and 35 mM; Figure 3). However, *NRXN2* −*SS4* was significantly downregulated in 7 mM compared with 0 mM ethanol (*F*_(df=8)_ = 22.6, *p*_ANOVA_ = 5.0 × 10^−2^; *p*_Dunnets_ = 3.3 × 10^−2^) but not when compared with 35 mM (*p*_Dunnets_ = 3.2 × 10^−1^). These results suggest that *NRXN2 −SS4* may be uniquely sensitive to low levels of ethanol in UnDiff IMR‐32 cells.

### 3.4 Broad *NRXN* Transcript Downregulation in Differentiated IMR32 Cells With Ethanol

**FIGURE 4:**
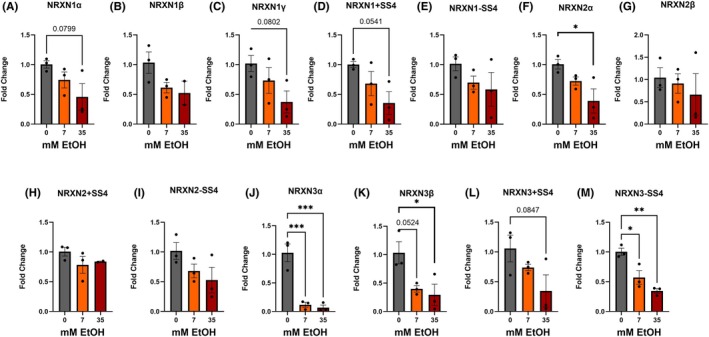
Differentiated IMR32 cells exhibit dose‐ and transcript‐specific downregulation of NRXN transcripts following ethanol exposure. IMR32 cells were differentiated for 4 days using all‐trans retinoic acid (tRA; 10 μM final concentration) prior to ethanol exposure. Cells were then cultured for 7 additional days in tRA‐containing medium supplemented with ethanol. Quantitative PCR analysis was used to assess transcript levels of 13 NRXN transcripts, including alternative splice variants at splice site 4 (SS4): (A) NRXN1α, (B) NRXN1β, (C) NRXN1γ, (D) NRXN1 +SS4, (E) NRXN1 −SS4, (F) NRXN2α, (G) NRXN2β, (H) NRXN2 +SS4, (I) NRXN2 −SS4, (J) NRXN3α, (K) NRXN3β, (L) NRXN3 +SS4, (M) NRXN3 −SS4—after exposure to 0, 7, or 35 mM EtOH. Bars show mean ± SEM; points denote biological replicates. *p* values reflect one‐way ANOVA with post hoc Dunnett's correction for multiple comparisons as described in methods; significant *p* values are represented by asterisks. Near significant *p* values (*p* < 0.10) are displayed numerically.

Following tRA‐induced differentiation, and contrarily to what we observed in the UnDiff IMR32 cells, ethanol treatment led to a significant downregulation of multiple NRXN transcripts (Figure 4). Although *NRXN2 −SS4* was not downregulated in Diff IMR32s (*F*_(df=8)_ = 2.4, *p*_ANOVA_ = 1.7 × 10^−1^), one‐way ANOVA analysis revealed *NRXN2α* (*F*_(df=8)_ = 5.4, *p*_ANOVA_ = 4.5 × 10^−2^), *NRXN3α* (*F*_(df=8)_ = 31.8, *p*_ANOVA_ = 6.0 × 10^−4^), *NRXN3β* (*F*_(df=8)_ = 6.3, *p*_ANOVA_ = 3.3 × 10^−2^), and *NRXN3 −SS4* (*F*_(df=8)_ = 18.1, *p*_ANOVA_ = 2.9 × 10^−3^) were significantly downregulated, whereas *NRXN1 +SS4* and *NRXN3 +SS4* expression levels nearly reached significance (*F*_(df=8)_ = 3.9, *p*_ANOVA_ = 8.1 × 10^−2^, and *F*_(df=8)_ = 3.0, *p*_ANOVA_ = 1.2 × 10^−1^). Following post hoc Dunnets correction for multiple comparisons, *NRXN3*α (*p*_Dunnets_ = 1.0 × 10^−3^) and *NRXN3 −SS4* (*p*_Dunnets_ = 1.4 × 10^−2^) were significantly reduced at 7 mM ethanol, whereas *NRXN3β* was nearly significantly (*p*_Dunnets_ = 5.2 × 10^−2^). At 35 mM, *NRXN2α* (*p*_Dunnets_ = 2.9 × 10^−2^) was significantly downregulated, along with a more dramatic decrease of *NRXN3α* (*p*_Dunnets_ = 7.0 × 10^−4^), *NRXN3β* (*p*_Dunnets_ = 2.9 × 10^−2^), and *NRXN3 −SS4* (*p*_Dunnets_ = 1.9 × 10^−3^). Of note, differential expression of several other transcripts nearly reached significance at 35 mM compared with 0 mM (*NRXN1α*: *p*_Dunnets_ = 7.9 × 10^−2^, *NRXN1γ*: *p*_Dunnets_ = 8.0 × 10^−2^, *NRXN1 +SS4*: *p*_Dunnets_ = 5.4 × 10^−2^, and *NRXN3 +SS4*: *p*_Dunnets_ = 8.4 × 10^−2^).

### 3.5 Differentiation‐Dependent Regulation of Neurexin Transcripts at Baseline and in Response to Ethanol

**FIGURE 5:**
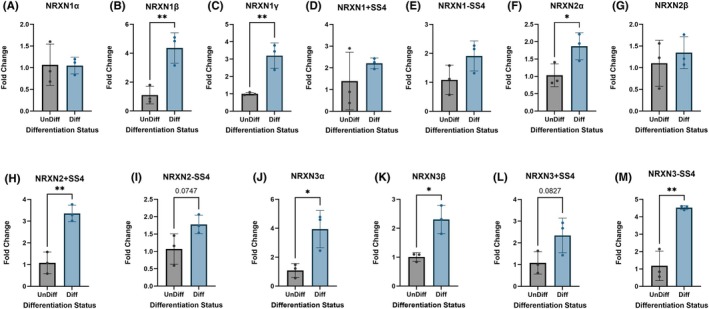
Differentiation modulates neurexin transcript levels in IMR‐32 cells in the absence of ethanol (0 mM). qPCR fold change (*y*‐axis) for neurexin transcripts and SS4 splice variants: (A) NRXN1α, (B) NRXN1β, (C) NRXN1γ, (D) NRXN1 +SS4, (E) NRXN1 −SS4, (F) NRXN2α, (G) NRXN2β, (H) NRXN2 +SS4, (I) NRXN2 −SS4, (J) NRXN3α, (K) NRXN3β, (L) NRXN3 +SS4, (M) NRXN3 −SS4. Bars show mean ± SEM; points denote biological replicates. Groups on the *x*‐axis are labeled by differentiation status (Diff = differentiated, UnDiff = undifferentiated). A Student's *t*‐test was performed, unless normal distribution was violated, in which case data were log‐transformed to achieve normality and statistically analyzed (displayed graphs were re‐transformed back into their native fold change values for visual consistency). Significant *p* values are displayed with asterisks; differences are interpreted at *α* = 0.05 after Sidak correction. Near significant *p* values are displayed numerically.

To determine whether there were differences in expression of NRXNs transcripts with differentiation in the absence of ethanol, we compared transcript expression at 0 mM ethanol (Figure 5) between UnDiff and Diff cells. Differentiation significantly increased basal expression of *NRXN1β* (*p*_*t*‐test_ = 9.8 × 10^−3^), *NRXN1γ* (*p*_*t*‐test_ = 6.6 × 10^−3^), *NRXN2α* (*p*_*t*‐test_ = 4.7 × 10^−2^), *NRXN2 +SS4* (*p*_*t*‐test_ = 3.2 × 10^−3^), *NRXN3α* (*p*_*t*‐test_ = 2.3 × 10^−2^), *NRXN3β* (*p*_*t*‐test_ = 1.2 × 10^−2^), and *NRXN3 −SS4* (*p*_*t*‐test_ = 2.5 × 10^−3^) (Figure 5B,C,F,H,J,K,M). We observed a trend of upregulation for *NRXN2 −SS4* (*p*_*t*‐test_ = 7.5 × 10^−2^), and *NRXN3 +SS4* (*p*_*t*‐test_ = 8.3 × 10^−2^) in Diff, whereas the rest showed no differences (Figure 5A,D,G).

**FIGURE 6:**
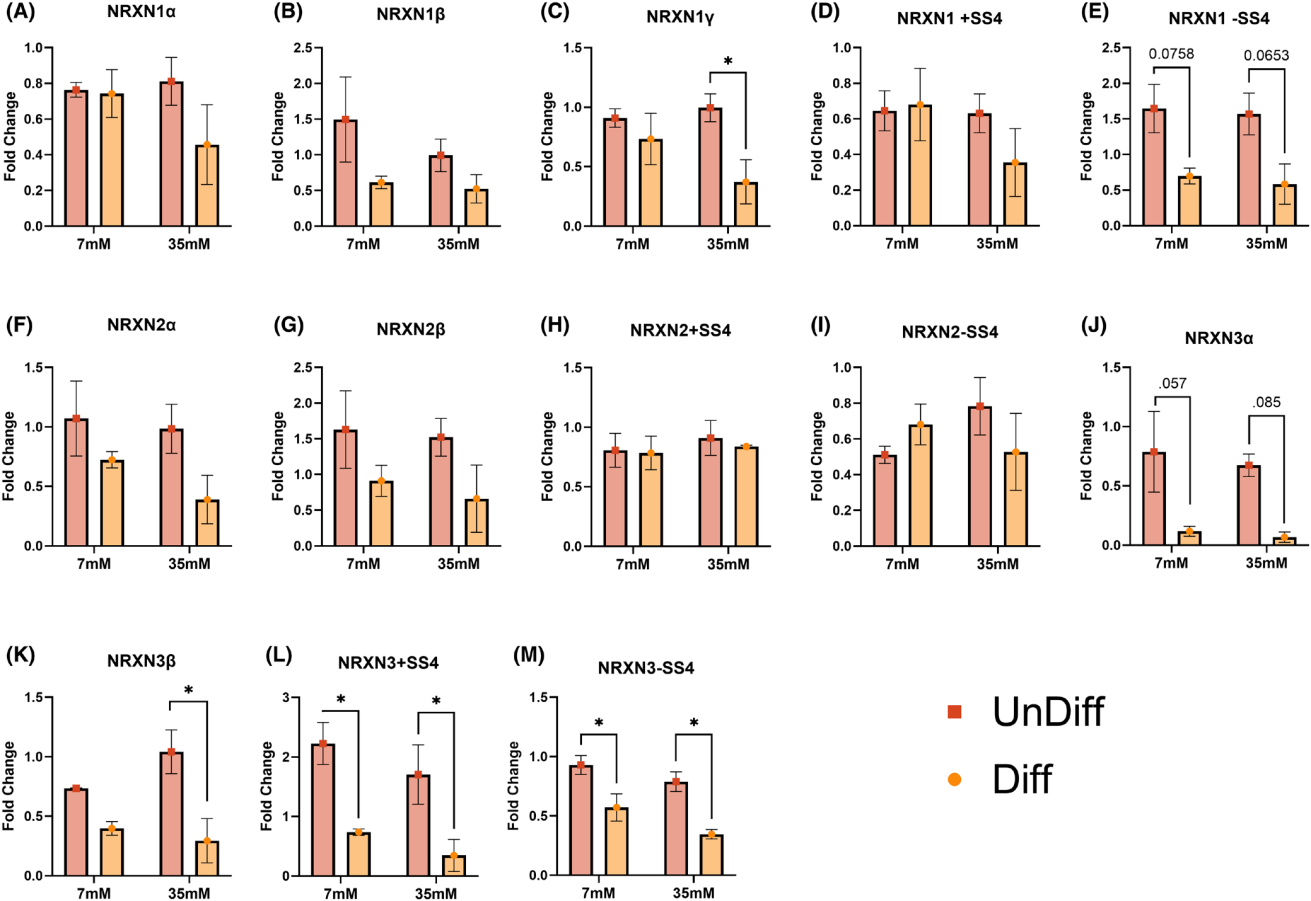
Differentiation and ethanol exposure modulate neurexin transcript levels in IMR‐32 cells. Quantitative PCR analysis was used to assess transcript levels of 13 NRXN transcripts, including alternative splice variants at splice site 4 (SS4): (A) NRXN1α, (B) NRXN1β, (C) NRXN1γ, (D) NRXN1 +SS4, (E) NRXN1 −SS4, (F) NRXN2α, (G) NRXN2β, (H) NRXN2 +SS4, (I) NRXN2 −SS4, (J) NRXN3α, (K) NRXN3β, (L) NRXN3 +SS4, (M) NRXN3 −SS4 after exposure to 0, 7, or 35 mM EtOH. Bars show mean ± SEM; Groups on the *x*‐axis are labeled by ethanol concentration and colors denote differentiation status (Diff = differentiated in light orange, UnDiff = undifferentiated in light red). Ordinary two‐way ANOVA with sources of variation ethanol concentration, differentiation status, and interaction (all effects tested with *F*_(1,9)_; residual df = 9) are reported in results. Sidak post hoc compare UnDiff versus Diff within each dose; adjusted *p* values are shown, and asterisks denote adjusted *p* < 0.05. For completeness, a main‐effects (additive) model was also fit to report marginal means; inferences were concordant with the full model.

Next, we investigated whether ethanol treatment would impact NRXNs expression between UnDiff and Diff IMR32s (Figure 6). After Sidak's correction for multiple comparisons, we found a robust main effect of differentiation at 35 mM for *NRXN1γ* (*F*_(df=8)_=6.3, *p*_2wayANOVA_ = 3.6 × 10^−2^; t_(df=8)_=2.8, *p*_sidak_ 4.9 × 10^−2^), *NRXN3*β (*F*_(df=7)_=12.8, *p*_2way_‐_ANOVA_ = 9.1 × 10^−3^; t_(df=7)_=3.7, *p*_sidak_ 1.5 × 10^−2^), *NRXN3 +SS4* (*F*_(df=8)_=18.1, *p*_2way_‐_ANOVA_ = 2.8 × 10^−3^; t_(df=8)_=2.9, *p*_sidak_ 4.1 × 10^−2^), and *NRXN3 −SS4* (*F*_(df=8)_=22.7, *p*_2way_‐_ANOVA_ = 1.4 × 10^−3^; t_(df=8)_=3.7, *p*_sidak_ 1.2 × 10^−2^). With nearly significant for *NRXN1 −SS4* (*F*_(df=8)_=12.7, *p*_2way_‐_ANOVA_ = 7.4 × 10^−3^; t_(df=8)_=2.6, *p*_sidak_ 6.5 × 10^−2^) and *NRXN3*α (*F*_(df=8)_=12.7, *p*_2way_‐_ANOVA_ = 7.3 × 10^−3^; t_(df=8)_=2.4, *p*_sidak_ 8.5 × 10^−2^). At 7 mM we found significant downregulation in Diff for *NRXN3 +SS4* at 7 mM (t_(df=8)_=3.2, *p*_sidak_ 2.7 × 10^−2^) and *NRXN3 −SS4* (t_(df=8)_=3.0, *p*_sidak_ 3.3 × 10^−2^). NRXN1 −SS4 was nearly significant at 7 mM (t_(df=8)_=2.5, *p*_sidak_ 7.7 × 10^−2^). In addition, several of these transcripts reversed directionality with differentiation; at 7 mM *NRXN3 +SS4* increased in UnDiff but decreased in Diff. At 35 mM, *NRXN3 +SS4* similarly switched from up‐ to downregulation in Diff relative to UnDiff (Figure 6). Together, these findings indicate that, during PV‐like differentiation, *NRXN3* transcripts show the most dramatic differences and are also the most sensitive to ethanol exposure.

### 3.6 Different NRXN Isoforms Are Downregulated in Differentiated IMR32 Cells After Ethanol Treatment

**FIGURE 7:**
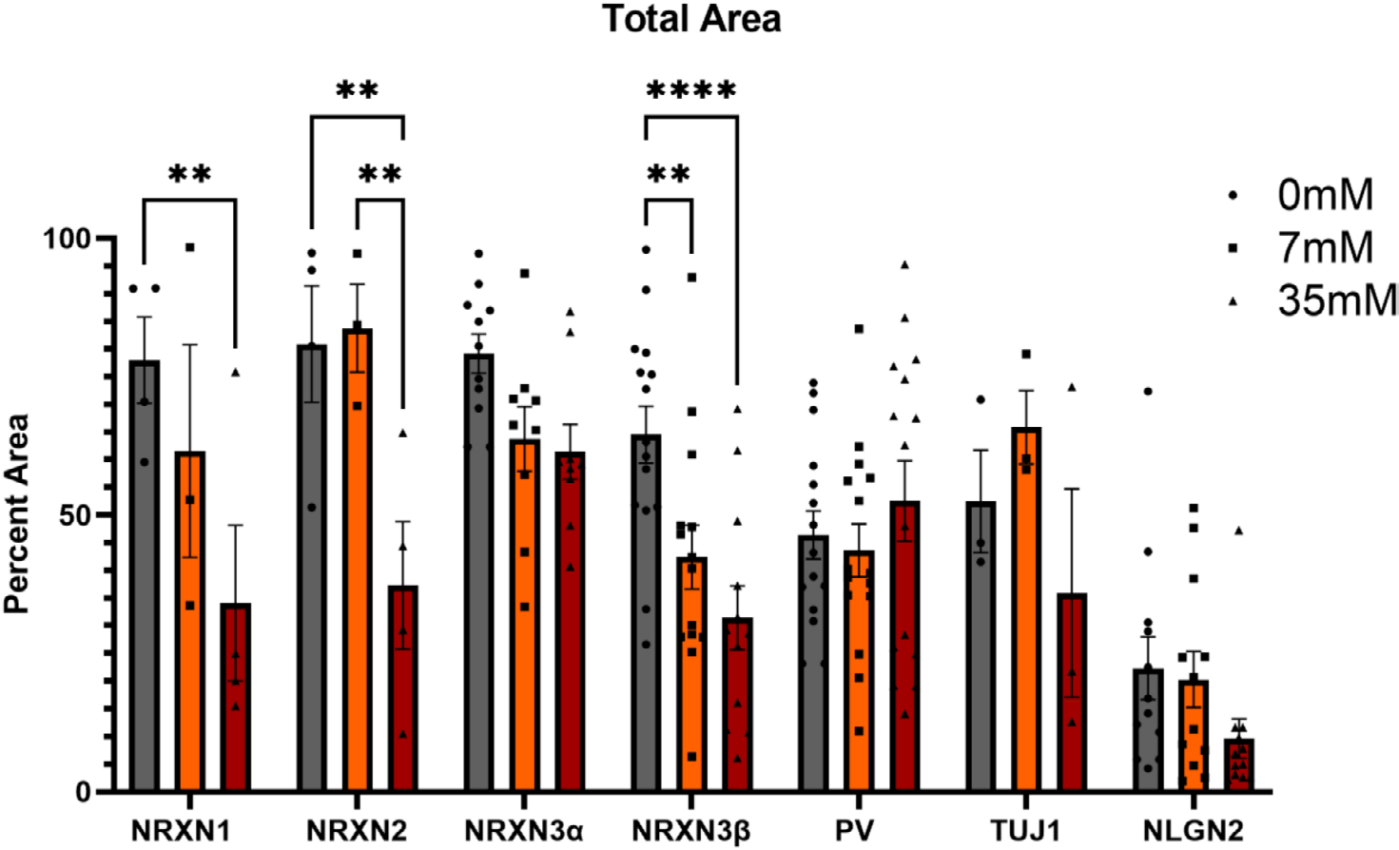
Ethanol exposure reduces expression of specific NRXN isoforms at the protein level in PV‐like IMR32 cells. Quantification of total immunoreactive area for NRXN isoforms (NRXN1, NRXN2, NRXN3a, and NRXN3β), structural neuronal markers (PV, TUJ1), and the postsynaptic adhesion protein NLGN2 after 7 days of ethanol exposure. IMR32 cells were first differentiated with all‐trans retinoic acid (tRA; 10 μM) for 4 days, followed by ethanol treatment at 0, 7, or 35 mM in continued tRA‐containing medium. Bars (mean ± SEM) show percent area staining positive for NRXN1 (*n* = 3–4 per ethanol concentration), NRXN2 (*n* = 3–4 per ethanol concentration), NRXN3α (*n* = 9–11 per ethanol concentration), NRXN3β (*n* = 12–15 per ethanol concentration), PV (*n* = 15 per ethanol concentration), TUJ1 (*n* = 3 per ethanol concentration), NLGN2 (*n* = 12 per ethanol concentration). Percent area = target protein‐positive area ÷ merged‐image area per field. Statistics: Two‐way ANOVA with factors Protein and Ethanol dose and their interaction. Post hoc testing used Tukey's comparisons 7 or 35 mM versus 0 mM within each protein; asterisks above brackets denote adjusted p values (*p < 0.05, **p < 0.01, ***p < 0.001, ***p < 0.0001). All multiple comparisons were corrected as indicated. Points represent biological replicates.

ICC analysis revealed significant main effects of changes in NRXN proteins (*F*_(6,161)_ = 21.7, *p*_2way_‐_ANOVA_ < 1.0 × 10^−4^) and ethanol dose (*F*_(2,161)=_15.5, *p*_2way_‐_ANOVA_ < 1.0 × 10^−4^) as well as a significant interaction (*F*_(12,161)_ = 2.6, *p*_2way_‐_ANOVA_ = 3.1 × 10^−3^), indicating dose effects differ by protein and isoform (for NRXN3). Although PV, TUJ1, and NLGN2 were not significantly altered with ethanol treatment, several NRXN isoforms were downregulated (Figure 7), indicating that ethanol specifically impairs expression of specific NRXN proteins without altering neuronal markers or this particular NRXN partner (NLGN2). For dose‐specific tests within each NRXN protein, following Tukey correction for multiple comparisons, NRXN3β showed the only significant decrease in expression (*p*_Tukey_ = 8.1 × 10^−3^) at 7 mM ethanol. At 35 mM ethanol, NRXN1 (*p*_Tukey_ = 5.4 × 10^−3^), NRXN2 (*p*_Tukey_ = 5.7 × 10^−3^) and most significantly NRXN3β (*p*_Tukey_ = < 1.0 × 10^−4^) showed a robust decrease when compared to controls, whereas NRXN3α showed a trend toward downregulation (Figure 7). For dose‐specific tests within each NRXN protein, NRXN2 had the only significant differences between 7 mM and 35 mM (*p*_Tukey_ = 6.5 × 10^−3^). These results support isoform‐ and dose‐specific downregulation of NRXNs by ethanol, with the NRXN3β showing the largest effect; as observed with the mRNA results (Figures 4 and 7).

### 3.7 Ethanol Alters Presynaptic–Postsynaptic Overlap Between NRXN3β and NLGN2 in Differentiated IMR32 Cells

**FIGURE 8:**
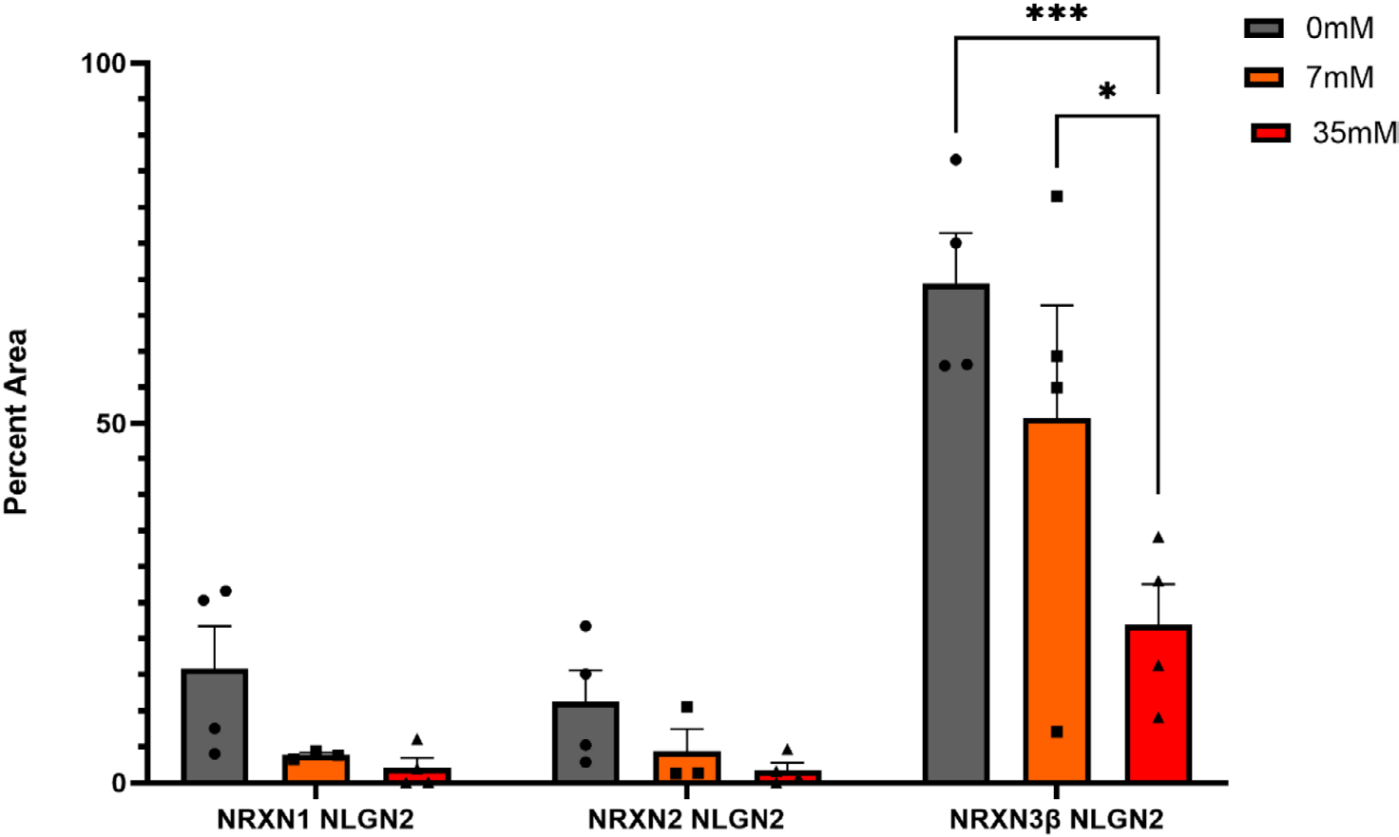
Ethanol disrupts NRXN3β–NLGN2 association in PV‐like IMR32 cells. IMR32 cells were differentiated with all‐trans retinoic acid (tRA; 10 μM) for 4 days, then exposed to 0, 7, or 35 mM ethanol for 7 days in continued tRA‐containing medium. Immunocytochemistry was used to assess the spatial overlap between NRXN isoforms (NRXN1, NRXN2, and NRXN3β) and the inhibitory marker neuroligin‐2 (NLGN2), displayed as the percentage of NLGN2‐positive area that overlapped with NRXN signal (overlap/NLGN2). *p* < 0.01 (**), *p* < 0.001 (***); two‐way ANOVA with post hoc comparisons. Points represent biological replicates (*n* = 3–4).

It is well documented that presynaptic NRXNs bind to postsynaptic NLGN2 (Zeppillo et al. 2024) to stabilize GABAergic synapses (Boxer and Aoto 2022). With tRA treatment we observed an enrichment in PV expressing neurons (Figure 2), and as NLGN2 is restricted to inhibitory synapses (Ali et al. 2020; Katzman and Alberini 2018; Zeppillo et al. 2024), we quantified the overlap between NRXNs and NLGN2 in our differentiated cultures. First, we quantified global NRXN and NLGN2 overlap across the entire ICC visual field (40×) not restricted to PV neurons, and observed a significant main effect but not interaction (Interaction: *F*_(4,25)_ = 2.5, *p*_2way‐ANOVA_ = 6.7 × 10^−2^; Protein: *F*_(2,25)_ = 34.58, *p*_2way_‐_ANOVA_ = < 1.0 × 10^−4^; Ethanol dose: *F*_(2,25)_ = 9.0, *p*_2way_‐_ANOVA_ = < 1.1 × 10^−3^), with a selective decrease in NRXN3β & NLGN2 overlap at 35 mM relative to 0 mM and 7 mM ethanol (0 mM: *p*_Tukey_ = 1.0 × 10^−4^, 7 mM: *p*_Tukey_ = 1.7 × 10^−2^), whereas NRXN1 & NLGN2 and NRXN2 & NLGN2 were not significant (Figure 8). It is important to note that the levels of NRXN3 were a lot higher than those of NRXN1 and NRXN2. We note that the NRXN3β & NLGN2 overlap metrics may not be indicative of direct binding; future work aiming at colocalization of these proteins with specific markers for pre and postsynaptic neurons is needed.

**FIGURE 9:**
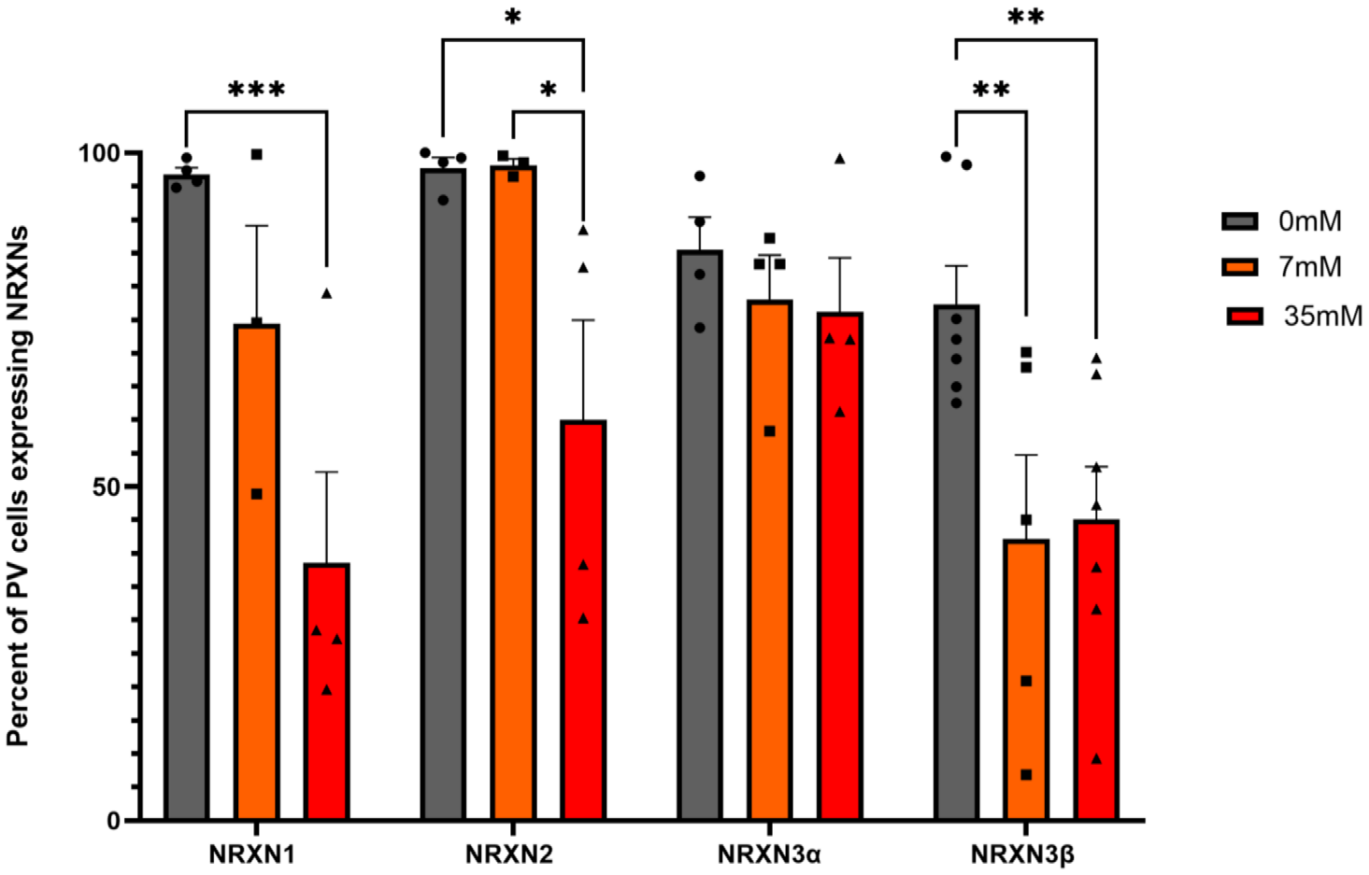
Ethanol exposure alters NRXN isoform localization in PV‐like IMR32 cells. IMR32 cells were differentiated with all‐trans retinoic acid (tRA; 10 μM) for 4 days, followed by 7 days of treatment with 0, 7, or 35 mM ethanol in continued tRA‐containing medium. Immunocytochemistry was used to examine the relationship between NRXN isoforms and PV‐positive cells. The percent of PV area containing NRXN signal, measured as (NRXN & PV)/PV was quantified for NRXN1 (*n* = 3–4), NRXN2 (*n* = 3–4), NRXN3α (*n* = 4) and NRXN3β (*n* = 5–7) signal within PV cells. Points represent biological replicates. *p < 0.05, **p < 0.01, ***p < 0.001; two‐way ANOVA with post hoc tests.

Given the critical role NRXNs play in PV function (Boxer and Aoto 2022; Boxer et al. 2021), we next focused on understanding ethanol‐associated changes in NRXN isoforms in PV‐positive cells. As described in methods, we measured the percent of PV area with NRXN signal (NRXN & PV divided by total PV area [(NRXN & PV)/PV]; Figure 9). A two‐way ANOVA (protein x ethanol dose) on [(NRXN & PV)/PV] showed significant main effects of ethanol dose and protein (ethanol dose: *F*_(2,41)_ = 14.4, *p*_2way_‐_ANOVA_ > 1.0 × 10^−4^; protein: *F*_(3,41)_ = 7.2, *p*_2way_‐_ANOVA_ = 5.0 × 10^−4^) without a significant interaction (*F*_(6,4)_ = 2.12, *p*_2way_‐_ANOVA_ = 7.2 × 10^−2^). Tukey post hoc (familywise *α* = 0.05) indicated that within PV‐positive cells, 35 mM ethanol significantly reduced NRXN1 (*p*_Tukey_ = 3.0 × 10^−4^), NRXN2 (*p*_Tukey_ = 2.2 × 10^−2^) and NRXN3β (*p*_Tukey_ = 8.3 × 10^−3^) (Figure 9) compared with controls. Uniquely, only NRXN3β showed a reduction at 7 mM ethanol (*p*_Tukey_ = 8.7 × 10^−3^) compared with 0 mM, and only NRXN2 showed a difference between 7 and 35 mM (*p*_Tukey_ = 3.3 × 10^−2^), as we observed for the non‐cell specific NRXN protein abundance (Figure 7). Similarly, and as observed for the non‐cell specific protein levels (Figure 7), NRXN3α did not change. These results indicate that ethanol exposure leads to an overall loss of NRXN signal, with PV‐positive neurons showing reduced expression of all three NRXNs (Figure 9).

## 4 Discussion

In this study, we used the human neuroblastoma IMR32 cells that were UnDiff or forced into mitotic arrest and differentiated toward cortical neuronal‐like cells with tRA treatment, to investigate how *NRXN* expression landscape adapts to physiologically relevant concentrations of ethanol. Use of 50‐100 mM ethanol for in vitro and ex vivo studies is common in the literature (Rath et al. 2022, Guadagnoli et al. 2016, Coleman Jr. et al. 2017, Crews et al. 2023, Crews et al. 2021, Lawrimore et al. 2019, Zou et al. 2022); however, we opted for modeling physiologically relevant concentrations based on the study by Thierauf‐Emberger et al. (2020). Specifically, we used 7 and 35 mM concentrations, which approximate BACs of ~0.044% and ~0.22%, respectively, to model low and high—but physiologically relevant—levels of intoxication. Our approach allowed us to examine early molecular adaptations to alcohol in a controlled, human‐neural system that captures ethanol dose‐sensitive transcriptional and protein/isoform adaptations, under the effects of ethanol levels that are detected in the brain of individuals consuming alcohol.

We first profiled the effects of differentiation on neurexin transcripts' expression. At baseline, and in the absence of ethanol, differentiation upregulated the expression of specific NRXN transcripts, including *NRXN1β, 1γ, NRXN2α, NRXN2 +SS4, NRXN3α, β, and NRXN3 −SS4*. Following ethanol exposure, several transcripts increased in UnDiff cells but decreased in Diff cells, particularly at higher ethanol concentrations. These results suggest that differentiation upregulates multiple *NRXN* transcripts, including members of the *NRXN3* class, whereas ethanol, particularly at intoxicating levels, prevents these in a splice‐ and transcript‐specific manner. Interestingly, some NRXN transcripts demonstrated ethanol dose‐dependent shifts in transcript abundance depending on the differentiation status. These results suggest that, in our in vitro system, NRXNs' transcripts adapt to the presence of ethanol in a dose‐dependent manner.

We next focused on the effects of different doses of ethanol on NRXN transcript profile in UnDiff or Diff cells. We found that ethanol (at any dose) had minimal effects on *NRXN* transcript expression in UnDiff cells. However, following differentiation, 35 mM ethanol exposure led to a downregulation of multiple *NRXN*s. These reductions occurred in the absence of cell death, suggesting that the observed downregulation of *NRXN* transcripts following high levels of ethanol is not attributable to cytotoxicity, but rather reflects specific transcriptional repression. Among the different NRXNs, *NRXN3* transcripts were the most impacted by ethanol. In addition to transcript‐level downregulation for *NRXN3α* and *β*, we also observed a significant reduction in the expression of *NRXN3 −SS4* (note that this SS is shared in *NRXN3α* and *β*, limiting our ability to discriminate both using qPCR). SS4 is a critical alternative exon that guides *NRXN* binding specificity to distinct postsynaptic ligands, including NLGNs and CBLNs (Aoto et al. 2013; Boucard et al. 2005), affecting long‐term potentiation (LTP) and long‐term depression (LTD) (Aoto et al. 2013). Seminal work showed that NRXN3 −SS4 promotes AMPAR‐mediated transmission (Aoto et al. 2013, Aoto et al. 2015), whereas other studies found that NRXNs +SS4 favors CBLN‐GluD signaling (Yasumura et al. 2012). However, some inhibitory functions of NRXN3α can proceed with limited dependence on SS4 (implicating other sites, such as SS2 and ligands like dystroglycan potentially facilitating this role [Trotter et al. 2023]). Taken together, a selective reduction of NRXN3 −SS4 by ethanol might diminish binding to NLGNs and shift the presynaptic binding toward binding partners that rely on the inclusion of SS4, such as CBLN1/2‐GluD (Uemura et al. 2010). This might ultimately disrupt normal AMPAR trafficking and excitatory/inhibitory balance (Dai et al. 2019; Liakath‐Ali and Sudhof 2021; Trotter et al. 2023). Further studies are needed to determine whether the levels of CBLN1/2 are also altered in this in vitro system and how synaptic transmission is affected by the ethanol‐associated adaptations in NRXN3.

As observed at the transcript level, ICC analysis confirmed that 35 mM ethanol selectively disrupted NRXN expression. Available antibodies for NRXN1 and NRXN2 do not distinguish between isoforms, but we observed consistent decreases at both the transcript and protein level for both. We note that the trend in *NRXN1* transcripts did not reach significance, although it was close. For NRXN3, we were able to use antibodies specific for NRXN3α and NRXN3β, confirming that NRXN3β was reduced at 7 and 35 mM across both assays. Although NRXN3α transcripts decreased at both ethanol concentrations, there was only an insignificant trend toward protein reduction after correcting for multiple comparisons. Together, these results reveal that ethanol exerts transcript/isoform‐ and dose‐specific effects on NRXN regulation, with most NRXNs showing transcriptional–protein concordance. Importantly, these effects are robust enough to be detected with two approaches that capture different molecules and at different cell type resolution. Specifically, bulk qPCR captures population‐wide transcriptional responses in a relatively heterogenous cell population, whereas ICC provides a higher degree of cellular resolution. The integration of these datasets therefore strengthens the robustness of our findings, revealing that differentiation and ethanol interact to reshape NRXN transcript and isoform profiles at multiple molecular levels in IMR32 cells.

PV interneurons play a critical role in modulating excitability of cortical pyramidal neurons (Druga et al. 2023; Hu et al. 2014; Packer and Yuste 2011; Ferguson and Gao 2018). Others have shown that NRXNs in PV interneurons, particularly NRXN3, can shift oscillatory dynamics and network excitability (Aoto et al. 2015; Boxer and Aoto 2022; Boxer et al. 2021; Luo et al. 2021). Thus, we sought to profile NRXNs adaptations within PV‐positive cells. We found that, although NRXN1, 2, and 3β levels decreased in PV‐positive cells, the loss of NRXN3β signal was notorious in PV cells. Direct empirical evidence for a specific NLGN2 and NRXN3β interaction is well documented, yet remains poorly understood. A particularly novel finding from our study is the ethanol‐induced disruption of the overlap of NRXN3β with NLGN2 at 35 mM of ethanol. Under control conditions (0 mM ethanol), NRXN3β accounted for a substantial fraction of NLGN2‐NRXN3β colocalized signal, compared with NLGN2‐NRXN1 or NLGN2‐NRXN2, consistent with its known role in direct binding to NRXN3β (Boxer and Aoto 2022; Bang and Owczarek 2013; Reissner et al. 2008). Our data suggest that at a high ethanol dose, reductions in these proteins, might lead to NRXN3β‐NLGN2 decoupling. Together with literature showing that ethanol perturbs synaptic adhesion pathways and inhibitory circuitry (Ferranti et al. 2022; Lovinger and Roberto 2025; McCool et al. 2003; Roberto et al. 2012), these observations support a potential working model in which intoxicating ethanol disproportionately reduces PV‐localized NRXN3 signal and diminishes NRXN3β–NLGN2 spatial apposition, potentially weakening inhibition of pyramidal neurons by PV interneurons and an overall hyperexcitatory microenvironment. Follow‐up studies are needed to fully understand the functional consequences of these alcohol‐linked molecular adaptations in the neurexin landscape to synaptic function.

In summary, integrating bulk transcription with PV‐focused and global ICC readouts argues that ethanol imposes a cell‐state‐dependent, isoform‐specific constraint on NRXN organization; with NRXN3β emerging as a particularly sensitive node connecting molecular and cellular changes. Specifically, our findings demonstrate that exposure of Diff IMR32 cells to physiologically relevant amounts of ethanol alters NRXN3β expression and co‐localization with specific postsynaptic partners in a splice‐dependent manner in cortical PV‐like interneurons. These results provide a mechanistic link between alcohol exposure and molecular disruptions in proteins known to drive inhibitory synapse organization and plasticity. By integrating clinically relevant ethanol dosing paradigms with high‐resolution spatial and molecular analysis in a human‐derived neural in vitro system, this work brings us a step closer in understanding the role of NRXNs in AUD.

Although IMR32 cells provide a flexible and reproducible platform for interrogating transcriptional and synaptic protein dynamics under controlled conditions, this in vitro system has several limitations that warrant careful interpretation of our findings. For instance, IMR32 neuroblastoma cells are derived from a 13‐month old male infant, and although we observed different NRXNs profiles between UnDiff and Diff cells, the Diff cells might still not fully recapitulate the maturation status of adult cortical neurons. In addition, and although tRA is effective at inducing cell cycle arrest and differentiation, the resulting cultures might be a mix of cells in different differentiation statuses (Chaudhari et al. 2017; Haussler et al. 1983; Sidell et al. 1983; Sivanantham et al. 2018). Thus, our transcriptomic analysis may be confounded by the heterogeneous composition of these cultures. Nonetheless, we observed robust ethanol‐associated decreases in several *NRXN* transcripts, and concordance in transcript and isoform profiles, indicating effects large enough to rise above this cellular heterogeneity. Although others have reported sex differences in neurexin function in rodents (Boxer and Aoto 2022; Boxer et al. 2021), use of the IMR32 limited the scope of our findings to males only. Lastly, we opted for a 7‐day ethanol treatment, in which cells were constantly exposed to ethanol in order to model chronic exposures. However, we recognize that this approach does not fully capture the patterns of ethanol intoxication displayed by humans or chronic intermittent ethanol (CIE) preclinical models, where repeated cycles of intoxication and withdrawal occur (Meinhardt and Sommer 2015). Future studies using co‐cultures or brain organoids generated from human induced pluripotent stem cells differentiated toward different neural cell types, or using postmortem cortical samples from individuals with AUD, or preclinical CIE models should be used to determine whether our findings are reproducible in more relevant biospecimens across males and females.

## Funding

This work was supported by the National Institute on Alcohol Abuse and Alcoholism (5T32AA007565‐30, 2T32AA007565‐31, 5T32AA007565‐32, R03 AA026092, R01 AA027552, R01 AA026278, and R01 AA032162) and the National Institutes of Health (1T32NS115704‐01A1).

## Conflicts of Interest

The authors declare no conflicts of interest.

## Data Availability

The data that support the findings of this study are available from the corresponding author upon reasonable request.
